# A new species of *Tipulodina* (Diptera, Tipulidae) from China, with description of the female internal reproductive system

**DOI:** 10.3897/zookeys.864.31755

**Published:** 2019-07-16

**Authors:** Guo-Xi Xue, Qiu-Lei Men, Jia Zhang, Qing Zhao, Nan Sheng, Hai-Xiao Wang, Ji-Feng Long

**Affiliations:** 1 School of Food and Bioengineering, Zhengzhou University of Light Industry, No. 5 Dongfeng Road, Zhengzhou, Henan 450002, China Zhengzhou University of Light Industry Zhengzhou China; 2 School of Life Sciences, Research Center of Aquatic Organism, Conservation and Water Ecosystem Restoration in Anhui Province, Anqing Normal University, Anqing, Anhui 246011, China Anqing Normal University Anqing China; 3 Administration of Nonggang National Nature Reserve of Guangxi, Longzhou, Guangxi, 532400, China Administration of Nonggang National Nature Reserve of Guangxi Longzhou China

**Keywords:** Crane flies, key, Nematocera, semen pump, Tipuloidea

## Abstract

A new species of the genus *Tipulodina* Enderlein, 1912, *Tipulodinabifurcata* Xue & Men, **sp. nov.** (Guangxi, South China) is described and illustrated. A key to the known species in China is provided. The morphological description of the female internal reproductive system of the new species is provided, which represents the first description for this genus.

## Introduction

The genus *Tipulodina* was established by Enderlein (1912) with the type species *Tipulodinamagnicornis* Enderlein, 1912 from Indonesia by original designation. It is a relatively large genus with 52 species worldwide, mainly restricted to the Oriental and Palaearctic regions (Oosterbroek 2018). Five species of this genus have been reported to occur in China before this study, four of which are distributed in southern China, and one from a northern part of the country (Oosterbroek 2018). *Tipulodina* can be separated from other tipulid genera by the following combination of characters: slender legs with femora and tibia having white rings (the tibia sometimes possessing two rings); wing transparent with a very short Rs and a dark pattern on apex; R_3_ reduced; gonocoxite generally with elongate appendage (Enderlein 1912, Young 1999).

A previously unknown species of *Tipulodina* was noticed while sorting and identifying crane fly specimens collected from Nonggang National Nature Reserve of Guangxi, China. In the present paper, the new species is described and illustrated, which represents the first record of a *Tipulodina* species from Guangxi. A key to separate the known species in China is given.

## Materials and methods

The specimens were collected using an insect net. All dissections and the photographs of the male body parts were performed using a SOIF XTZ-E stereo microscope (SOIF, Shanghai, China). The hypopygium of the male and ovipositor of the female were dissected in distilled water with the aid of two very fine needles, scissors and fine-tipped tweezers. and macerated in 10% NaOH for one hour in a 50 °C water bath. The structures were then observed and illustrated in glycerin under the stereo microscope. Body length measurements are from the vertex of head to the tip of the hypopygium. All measurements were made in millimeters (mm) with the aid of a digital caliper. The terminology and methods of description and illustration follow those of Alexander and Byers (1981), Frommer (1963) and de Jong (2017). The type specimens are deposited in the Systematics and Evolution Laboratory, School of Life Sciences, Anqing Normal University, Anhui Province, P. R. China.

The first and corresponding authors were responsible for the taxonomic portion of this paper, and are therefore the authors of the new species. The key was principally constructed from descriptions in the literature without examination of the types or other specimen of most of these species, and should be considered preliminary.

## Taxonomy

### Tipulodina bifurcata

Taxon classificationAnimaliaDipteraTipulidae

Xue & Men sp. nov.

e5123a9c-6034-4a78-94f8-ca8506225cc3

http://zoobank.org/E14D124D-1789-4326-9C08-7B896BE3CA95

Figs 1–8 , 9–16 , 17–20 , 22–24

#### Material examined.

**Holotype**: male. **CHINA**: Guangxi, Longzhou County, Nonggang National Nature Reserve, 9 April 2018, leg. Guoxi Xue. **Paratype**: 1 female, same data to holotype.

#### Diagnosis.

The only male specimen of *Tipulodina* with the following combination of characters: antenna with scape white on basal two thirds, black on apical third, remaining segments black; prescutum with three brown stripes, median one divided by a narrow black vitta; wing transparent, stigma black, wing tip suffused with black on outer ends of cells r_1_, r_4_ and r_5_; fore and mid tibia with one white ring near apex, hind femora with two white rings; tergite nine shallowly emarginated on hind margin, densely covered with black setae; appendage of gonocoxite with a long horn-shaped rod, curved, black, sharply acute at apex, fringed with long yellow setae, with black bifurcate process inserted basally.

#### Description.

**Male**. Length: body 13.1 mm (not including antenna, n = 1); wing 12.3 mm (n = 1); antenna 3.3 mm (n = 1).

**Figures 1–8.**
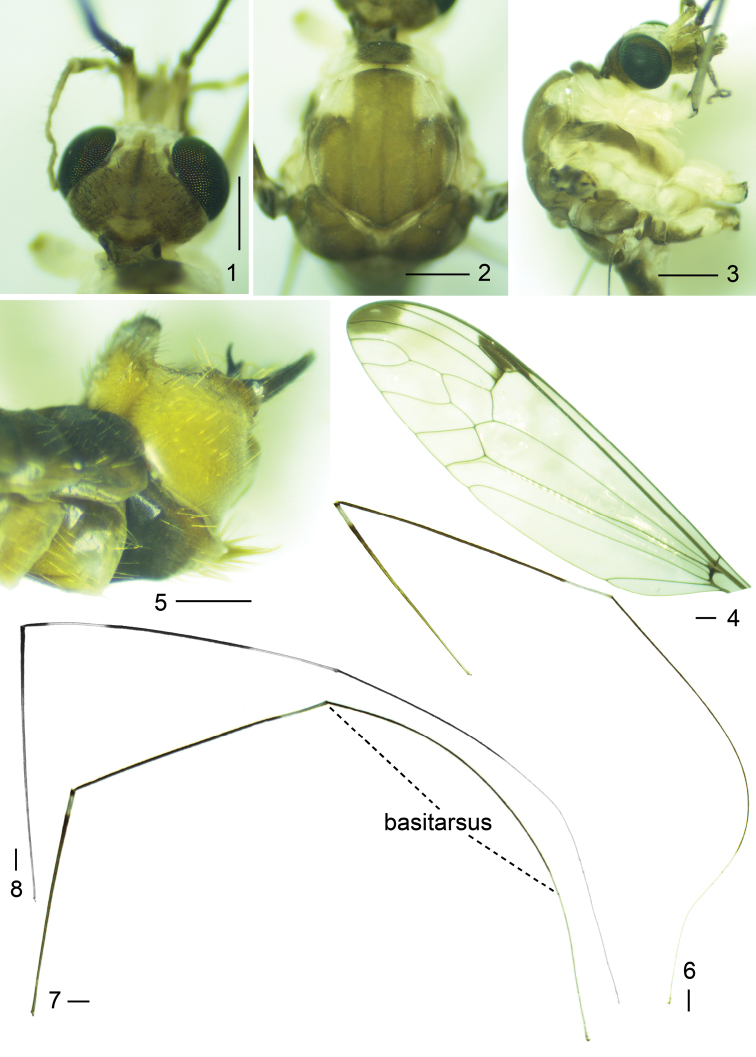
*Tipulodinabifurcata* Xue & Men, sp. nov. **1** head, dorsal view **2** thorax, dorsal view **3** thorax, lateral view **4** wing **5** hypopygium, lateral view **6** fore leg **7** middle leg **8** hind leg. Scale bars: 0.5 mm (**1–5**); 1.0 mm (**6–8**).

***Head.*** Rostrum white with white nasus, densely covered with black setae. Eye black. Occiput light brown (Fig. 1). Vertex light brown, medially with narrow pale line (Fig. 1). Antenna: bent backward extended beyond base of first abdominal segment; scape white on proximal two thirds, gradually changed to black on distal third, cylindrical, slightly expanded at apex; pedicel black, very short; flagellum entirely black, first flagellomere longest, subequal in length to scape, remaining segments progressively shortened, bases of each flagellomere with five black verticils, of which longest one significantly shorter than its corresponding flagellomere, surface of each flagellomere densely covered with short black setae. Palpus white, three basal segments distinctly thicker than apical segment.

***Thorax.*** Pronotum white laterally, black on middle third (Fig. 2). Prescutum white with three brown stripes; median one with lateral margins parallel, anterior margin suffused with black, black median vitta dividing by median stripe into two parts; lateral stripes half the length of median one, their apices also black (Fig. 2). Scutum white anteriorly and medially, with two light brown markings connected to each other, upper one distinctly smaller than lower one. Scutellum entirely white (Fig. 2). Postnotum wholly dark brown. Pleuron white, with two brown stripes, anterior stripe extending throughout the anterior spiracle, anepisternum and katepisternum, posterior stripe throughout posterior spiracle, laterotergite and hind coxa (Fig. 3). Legs very slender, coxae and trochanters white, the latter narrowly margined with black at apex (Figs 6–8); fore and mid legs with femora brown at base, gradually changed into black, with white ring near apex, tibiae black with white ring at apex, basitarsi black on proximal four fifths, white on distal fifth, remaining segments white (Figs 6, 7); hind leg with femur brown at base, brown becoming darker on distal portion, tibia black with two white rings, basal one slightly shorter than apical one, the latter reaching end of tibia, basitarsus black on basal four fifths, white on apical fifth, remaining tarsomeres white (Fig. 8). Halter with stem brown, knob darker. Wing glassy and transparent, stigma black, wing tip tinged with black on outer ends of cells r_1_, r_4_ and r_5_, light spot situated in middle of black region of cell r_4_; discal cell transparent, broadened. Venation: Rs very short, R_3_ reduced, petiole of cell m_1_ slightly shorter than discal cell, the latter slightly longer than cell m_1_ (Fig. 4).

**Figures 9–16.**
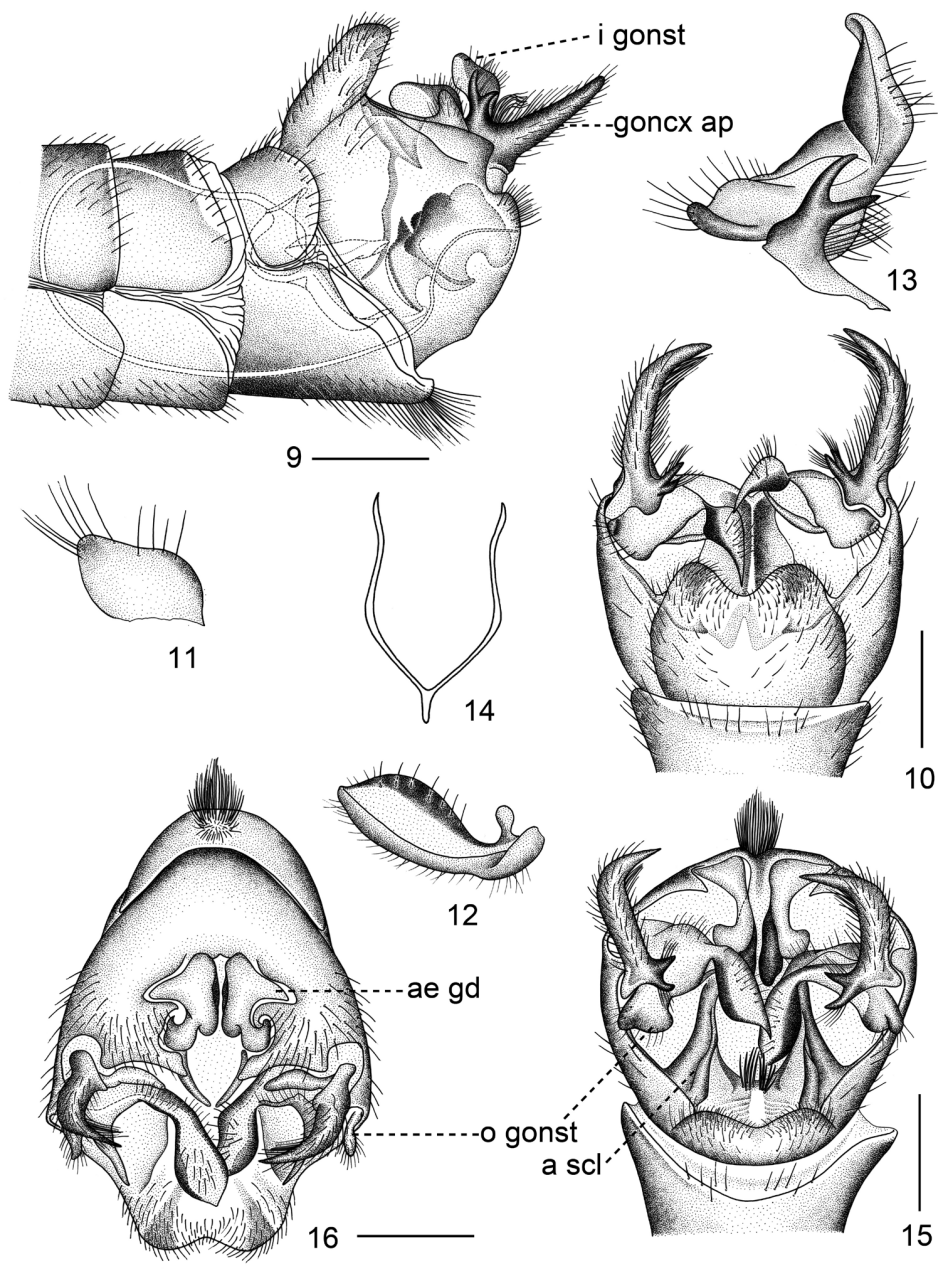
*Tipulodinabifurcata* Xue & Men, sp. nov. **9** hypopygium, lateral view **10** hypopygium, dorsal view **11** outer gonostylus, lateral view **12** inner gonostylus, lateral view **13** inner gonostylus and bifurcate process on appendage of gonocoxite, lateral view **14** genital bridge, dorsal view **15** hypopygium, caudal view **16** hypopygium, caudal view. Abbreviations: a scl = anal sclerite; ae gd = aedeagal guide; goncx ap = appendage of gonocoxite; i gonst = inner gonostylus; o gonst = outer gonostylus. Scale bars: 0.5 mm.

**Figures 17–24.**
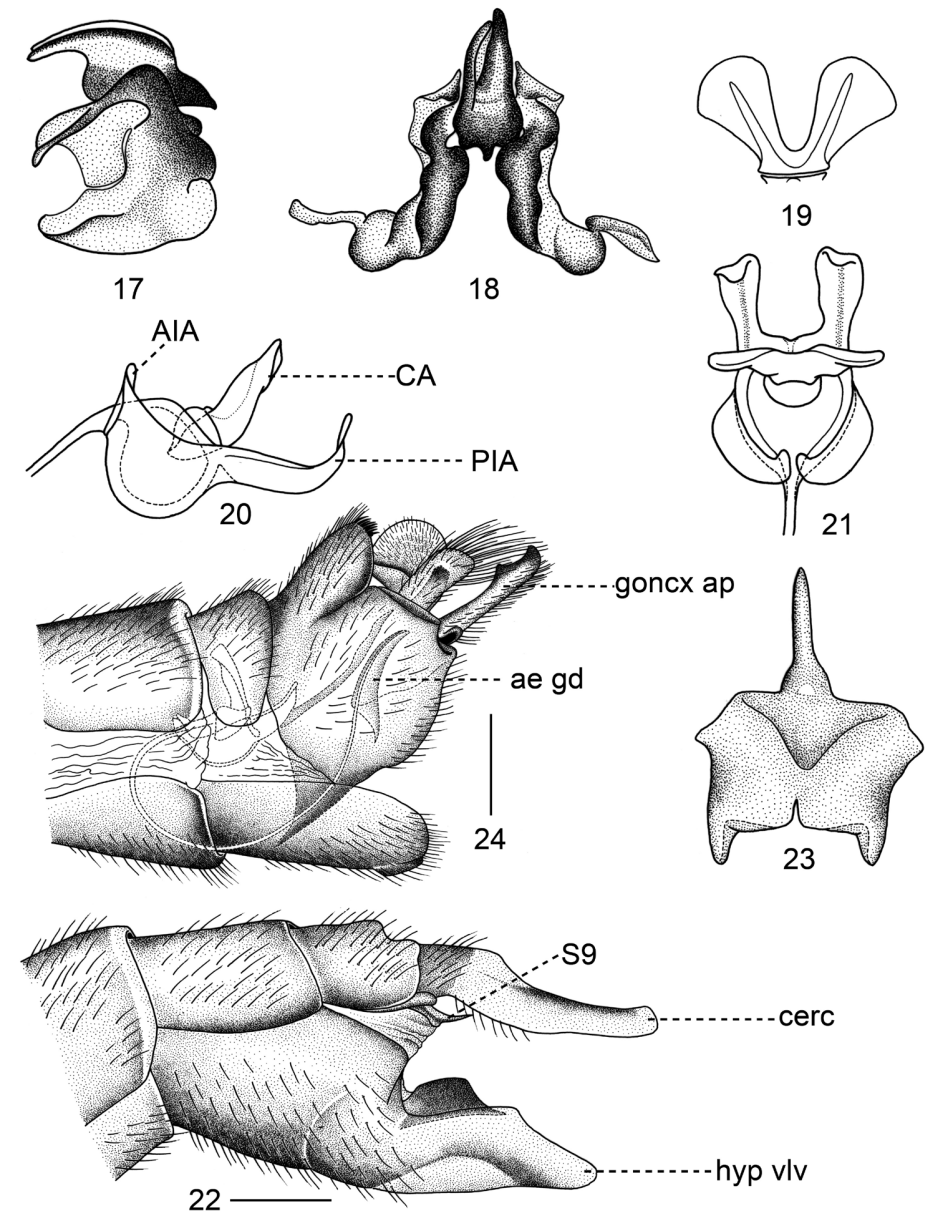
**17–23***Tipulodinabifurcata* Xue & Men, sp. nov. **17** aedeagal guide, lateral view **18** aedeagal guide, dorsal view **19** compressor apodeme, dorsal view **20** semen pump, lateral view **21** semen pump, dorsal view **22** ovipositor, lateral view **23** sternite nine, dorsal view **24***Tipulodinaxyris*, hypopygium, lateral view. Abbreviations: ae gd = aedeagal guide; AIA = anterior immovable apodeme; CA = compressor apodeme; cerc = cercus; goncx ap = appendage of gonocoxite; hyp vlv = hypogynial valve; PIA = posterior immovable apodeme; S9 = sternite 9. Scale bars: 0.5 mm.

***Abdomen.*** Tergite 1 yellowish brown, dark on both anterior corners, tergite 2 yellowish brown, encircled in yellow medially and ringed with yellow at base, tergites 3 and 4 yellowish brown, narrowly suffused with yellow at base, tergites 5 to 8 entirely brown, all tergites narrowly bordered with black on lateral and hind margins, sternites yellowish brown; hypopygium yellow (Fig. 5). Hypopygium with tergite nine and sternite nine almost separated from each other, only fused at base (Figs 5, 9, 10, 12). Tergite nine U-shaped and emarginated at hind margin, densely covered with small black setae (Figs 10, 15); median area extended with pair of anal sclerites, between them with membranous area invisible from above, a few long yellow setae placed in the middle of this membranous area (Fig. 15). Appendage of gonocoxite bearing two parts: a big horn-shaped rod tapered to sharply acute and curved black apex, fringed with many long and yellow setae on lateral margin, and black bifurcated process originating from the base of the horn-shaped rod (Figs 5, 9, 10, 15, 16). Outer gonostylus small oval lobe, obtuse apically, tightly connected to base of inner gonostylus, bearing several long setae on outer margin (Figs 10, 11, 15, 16). Inner gonostylus, a fusiform lobe, with long setae on inner side, edged in black on both margins (Figs 12, 13, 15, 16). Sternite nine broader than tergite nine, deeply concave on posterior border (Fig. 16). Sternite eight shallowly concave on posterior border, medially with a group of long setae pointing caudally (Figs 5, 9, 15, 16). Genital bridge connected with base of gonocoxite, S-shaped, converged posteriorly into small common stem (Fig. 14). Aedeagal guide broad basally, narrowed apically, curved caudad, separated at apical half, with pair of lateral arms with inner margins expanded and variegated with black (Figs 9, 17, 18).

***Semen pump.*** Compressor apodeme divided into two lobes by V-shaped notch, each lobe expanded apically with median ridge (Fig. 19). Posterior immovable apodeme with two arms elongated and curved dorsally (Figs 20, 21). Anterior immovable apodeme flattened and short, rounded in dorsal view (Figs 20, 21). Aedeagus elongated, tubular, thick basally, gradually narrowed to apex, more than 5.0 times longer than length of semen pump (Fig. 9).

#### Female.

Length: body 15.3 mm (not including antenna, n = 1); wing 15.4 mm (n = 1); antenna 2.5 mm (n = 1).

***Coloration.*** General coloration of head, thorax, and abdomen similar to male.

**Figures 25–28.**
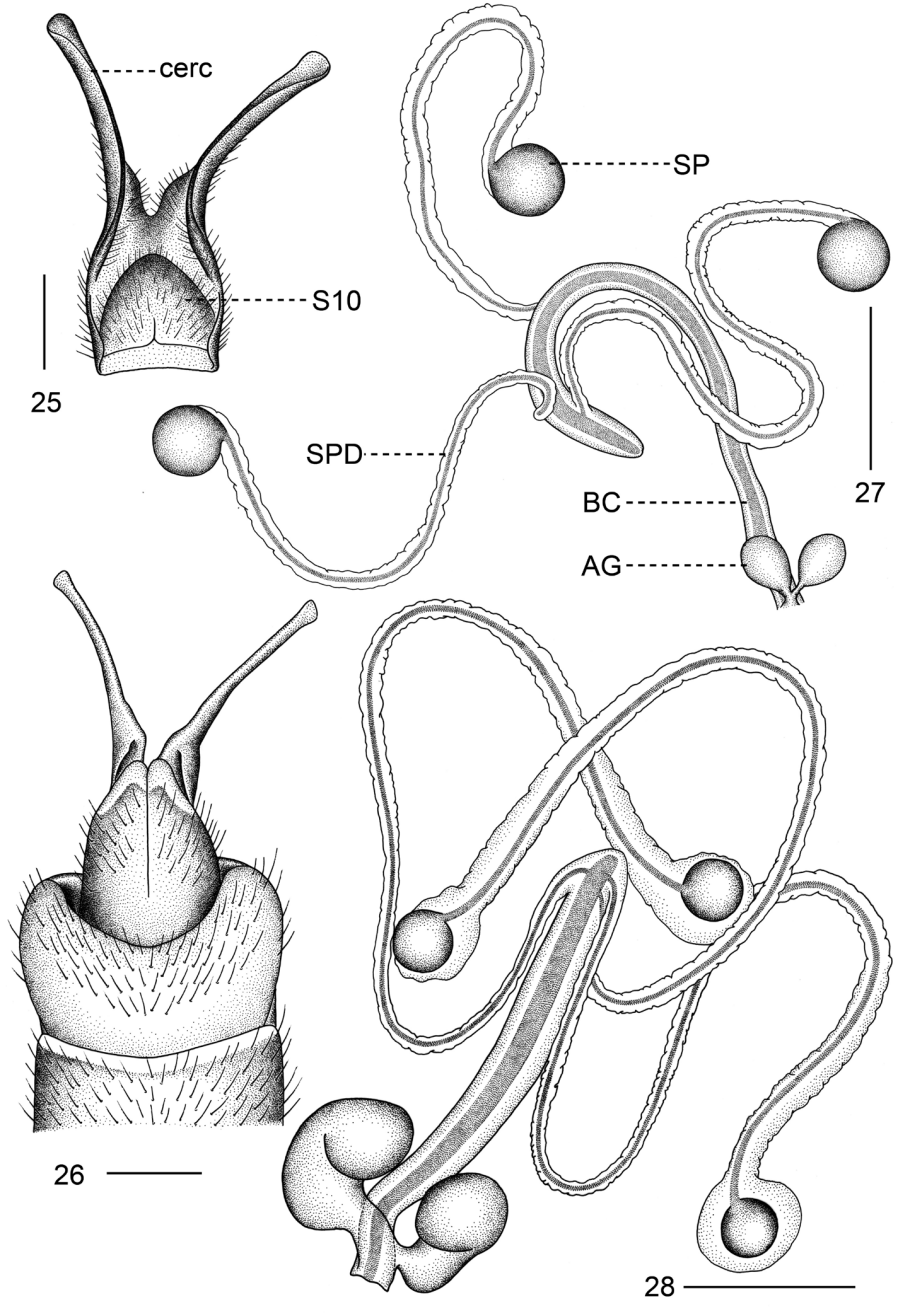
**25–27***Tipulodinabifurcata* Xue & Men, sp. nov. **25** cerci and sternite ten, ventral view **26** ovipositor, dorsal view **27** female internal reproductive system **28***Tipulodinaxyris*, female internal reproductive system . Abbreviations: AG = accessory gland; BC = bursa copulatrix; cerc = cercus; SP = spermatheca; SPD = spermathecal duct; S10 = sternite 10. Scale bars: 0.5 mm.

***Ovipositor.*** Yellowish brown in general. Sternite nine broad basally, acute apically; tergite nine broad, longer than tergite ten in lateral view (Fig. 22); sternite ten obtuse apically, densely covered with small setae, tergite ten broad at base, gradually narrowed to apex, the latter divided by suture (Figs 22, 25, 26). Cercus long, slight curved, widened at basal fourth, narrowed towards apex, slightly expanded apically, surpassing end of hypogynial valve (Figs 22, 25, 26). Hypogynial valve distinctly broader than cercus, rounded apically, broadened medially, bearing a black lobe on dorsal margin (Fig. 22).

***Female internal reproductive system.*** Consisting of pair of accessory glands, bursa copulatrix, and three spermathecae with respective spermathecal duct (Fig. 27). Bursa copulatrix relatively elongate and narrow, rounded apically (Fig. 27). Accessory gland arising from base of bursa copulatrix, as pair of oval and swollen balls, terminating in small common stem (Fig. 27). Spermatheca spherical, black, bigger than the accessory gland (Fig. 27). Spermathecal duct slender, distinctly narrower than bursa copulatrix, flexible, generated from distal part of bursa copulatrix; connection points of three spermathecal ducts with bursa copulatrix not at same level (Fig. 27).

#### Distribution.

China (Guangxi).

#### Remarks.

The new species is generally similar to *Tipulodinaxyris* (Alexander, 1949) by its colorations of the wing and legs (Alexander 1949, Men et al. 2016). The new species can be separated from related species by the appendage of the gonocoxite possessing a basal horn-shaped rod and a bifurcate process (not present in *T.xyris*), by the hind leg with basitarsus black on basal four fifths (hind leg with basitarsus black at basal third in *T.xyris*), by the aedeagus more than 5.0 times longer than the length of semen pump (about 3.0 times longer than the length of its semen pump in *T.xyris*) and by the shape of the aedeagal guide (Figs 9, 24). This new species also differs from *T.xyris* in organs of female internal reproductive system, including bigger spermathecae and accessary gland, and a narrower bursa copulatrix (Figs 27, 28).

#### Etymology.

The specific epithet is an adjective derived from the Latin *furcata* meaning forked, with the Latin prefix *bi*, referring to the presence of a bifurcate process on the appendage of the gonocoxite.

### Key to Chinese *Tipulodina* species (Fig. 29)

**Table:** 

1	Hind tibia with single white ring (Alexander 1923: 76)	***T.taiwanica* Alexander, 1923** (Taiwan, Xinzhu)
–	Hind tibia with two white rings	**2**
2	Middle femur with white ring immediately before apex (Alexander 1949: 524; Men et al. 2016: 96)	**5**
–	Middle femur without white ring	**3**
3	Hind tarsus with apical 3 segments not white (Alexander 1936: 175; Yang 1999: 39)	***T.hopeiensis* (Alexander, 1936)** (Hebei, Tangshan, Qingdongling)
–	Hind tarsus with apical 3 segments white	**4**
4	Flagellum entirely black; cell sc and stigma black (Yang 1999: 39)	***T.jigongshana* Yang, 1999** (Henan, Jigongshan)
–	Flagellum bicolored; cell sc and stigma dark brown (Alexander 1938: 444, 445)	***T.cantonensis* (Alexander, 1938)** (Guangdong, Honam Island = Haizhu District)
5	Appendage of gonocoxite without a bifurcate process	***T.xyris* (Alexander, 1949)** (Anhui, Huangshan, Tangkou; Fujian, Wuyishan)
–	Appendage of gonocoxite with a bifurcate process on lateral side	***T.bifurcata* Xue & Men, sp. nov.** (Guangxi, Nonggang National Nature Reserve)

**Figure 29.**
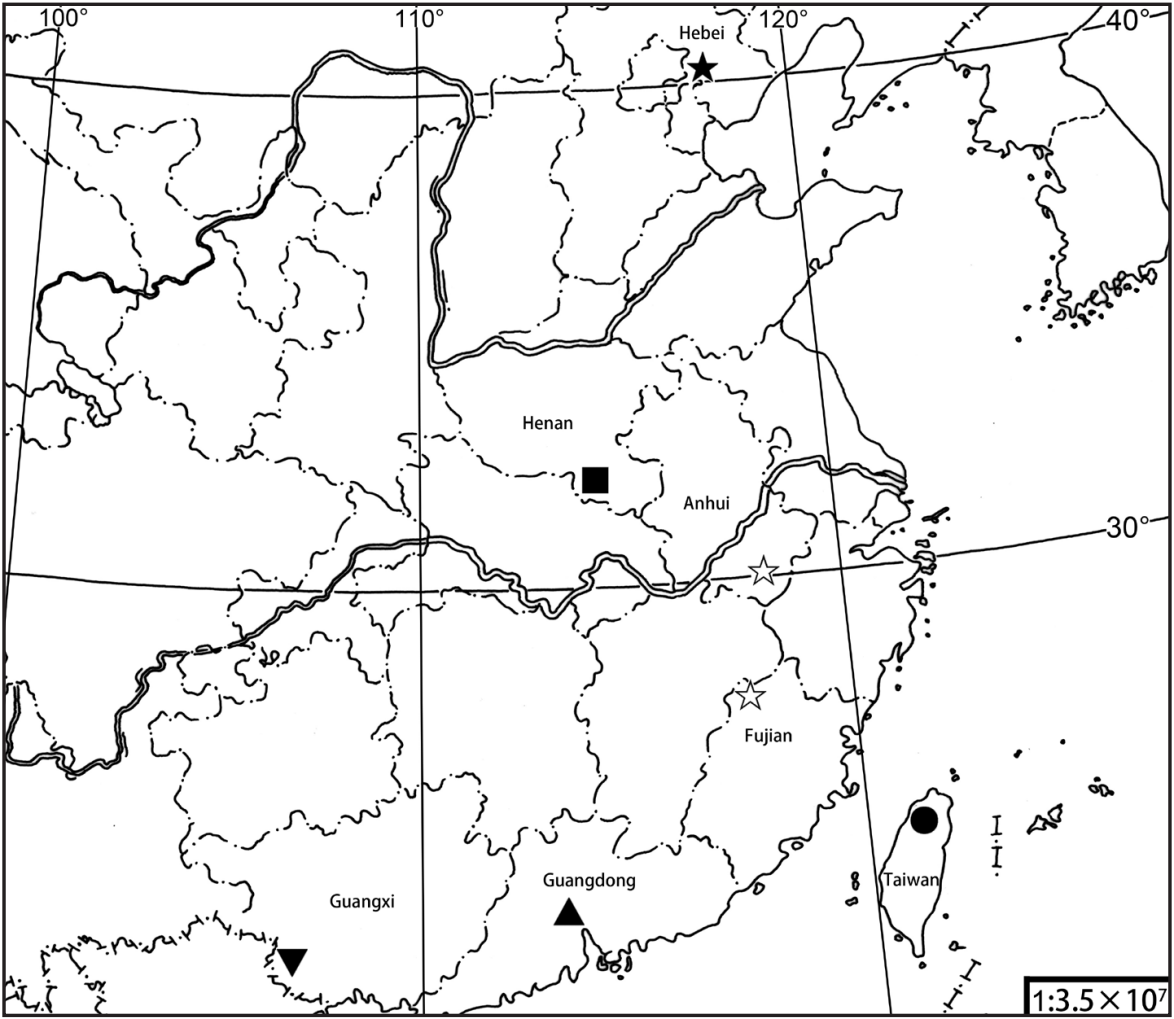
Geographic distribution of *Tipulodina* species in China. Key to symbols: *T.taiwanica* (circle), *T.hopeiensis* (black star), *T.jigongshana* (square), *T.cantonensis* (up-pointing triangle), *T.xyris* (white star), *T.bifurcata* (down-pointing triangle).

## Supplementary Material

XML Treatment for Tipulodina bifurcata
